# Engineered Skin Substitute Regenerates the Skin with Hair Follicle Formation

**DOI:** 10.3390/biomedicines9040400

**Published:** 2021-04-08

**Authors:** Jinmei Wang, Xiaoxiao Wang, Jundong Xie, Bin Yao, Miaohua Mo, Danjun Ma, Chen Huang, Renhe Xu, Xiaobing Fu, Edward E. Tredget, Yaojiong Wu

**Affiliations:** 1 School of Mechanical Engineering, Dongguan University of Technology, 1 Daxue Rd, Dongguan 523830, China; wangjinmei067@163.com (J.W.); madanjun@hotmail.com (D.M.); 2 iBHE, Shenzhen International Graduate School, Tsinghua University, Shenzhen 518055, China; wangxx_2005@126.com (X.W.); xiejundonggg@sina.com (J.X.); hongyaobin1212@163.com (B.Y.); momh@gdmu.edu.cn (M.M.); 3 State Key Laboratory of Chemical Oncogenomics, Shenzhen International Graduate School, Tsinghua University, Shenzhen 518055, China; 4 Tsinghua-Berkeley Shenzhen Institute (TBSI), Tsinghua University, Shenzhen 518055, China; 5 Guangdong Provincial Key Laboratory of Cell Microenvironment and Disease Research, Shenzhen Key Laboratory of Cell Microenvironment, Department of Biology, Southern University of Science and Technology, Shenzhen 518055, China; 6 Department of Cell Biology and Genetics, School of Basic Medical Sciences, Xi’an Jiaotong University Health Science Center, Xi’an Jiaotong University, Xi’an 710061, China; hchen@mail.xjtu.edu.cn; 7 Faculty of Health Sciences, University of Macau, Macau 999078, China; renhexu@umac.mo; 8 Wound Healing and Cell Biology Laboratory, Institute of Basic Medical Science, Chinese PLA General Hospital, Beijing 100088, China; fuxiaobing@vip.sina.com; 9 Stem Cell and Tissue Regeneration Laboratory, The First Affiliated Hospital, Chinese PLA General Hospital, Beijing 100084, China; 10 Wound Healing Research Group, Department of Surgery, University of Alberta, Edmonton, AB T6G2B7, Canada

**Keywords:** skin derived precursors, hair follicle neogenesis, engineered skin substitute

## Abstract

Currently, engineered skin substitutes (ESS) are unable to regenerate cutaneous appendages. Recent studies have shown that skin-derived precursors (SKPs), which are extensively available, have the potential to induce hair follicle neogenesis. Here, we demonstrate that ESS consisting of culture-expanded SKPs and epidermal stem cells (Epi-SCs) reconstitute the skin with hair follicle regeneration after grafting into nude mice. SKPs seeded in a C-GAG matrix proliferated and expressed higher levels of hair induction signature genes—such as *Akp2*, *Sox2*, *CD133* and *Bmp6*—compared to dermal fibroblasts. Moreover, when ESS prepared by seeding a mixture of culture-expanded murine SKPs and human adult Epi-SCs into a C-GAG matrix was grafted into full-thickness skin wounds in nude mice, black hairs were generated within 3 weeks. Immunofluorescence analysis showed that the SKPs were localized to the dermal papillae of the newly-formed hair follicle. Our results indicate that SKPs can serve as the hair-inductive cells in ESS to furnish it with hair genesis potential

## 1. Introduction

Skin, which is the largest organ of the body, acts as a barrier against infections and water loss, and has several homeostatic/sensory functions which are important to health. In severe skin defects due to burns or other causes, split-thickness skin autografts are often used to treat patients. However, suitable donor sites for autografts are often unavailable in many cases, and therefore skin substitutes are the preferred treatment option. Engineered skin substitutes (ESS) containing autologous fibroblasts and keratinocytes seeded on an artificial extracellular matrix made of collagen-glycosaminoglycan (C-GAG) are the most promising product due to their favorable clinical results in wound healing [1,2,3]. The ESS is able to form a functional epidermal barrier after transplantation onto the wound bed, but do not generate epidermal appendages, e.g. hair follicles, sebaceous glands, and sweat glands; thereby, the structure and function of the skin are not fully restored [4,5,6]. An ESS capable of regenerating the epidermis and the epidermal appendages has been a challenge.

The first important challenge of engineering an ESS is finding appropriate cell sources and providing large cell quantities. Skin appendages develop from a single layer of multipotent embryonic progenitors during skin morphogenesis, and extend deeply into the dermis [7]. The initiation of de novo hair follicles may be the first signal from the dermis during skin development [8,9,10]. Previous studies have shown that dermal papilla (DP) cells from hair follicles have the ability to induce hair follicle regeneration. However, the limitations of DP cells, such as the small amount, the limited availability, and the difficulty in maintaining their hair-follicle–inductive ability in in vitro culture, prevent them from being widely used in ESS [11,12,13]. Interestingly, recent studies indicate that multipotent skin-derived precursors (SKPs)—present in the dermis of neonatal or adult human and mouse skin—have been shown to induce hair follicle neogenesis when transplanted, indicating their potential applications in hair follicle regeneration and ESS [14,15].

Scaffolds, an essential component in tissue engineering, mimic some critical aspects of the extracellular matrix, and modulate stem cells to survive, differentiate, and form functional tissue structures [16,17,18]. Many of the natural and synthetic biomaterials, and many different technologies—such as micro- and nanotechnologies and additive manufacturing—have been integral for advancing the field of tissue engineering scaffold [19,20]. Of the many scaffold materials being investigated, the C-GAG porous scaffold remains to the most reliable scaffold for ESS on patients due to its overall properties in outstanding biological function, appropriate degradation speed, and sufficient physical strength, though numerous studies (including ours) have attempted to find a better one [21,22]. Moreover, the survival and hair follicle-inductive ability of SKPs in C-GAG have not been studied.

In this work, we found that culture-expanded murine SKPs were viable and proliferating in an C-GAG scaffold, resulting in an increase in the number of SKPs. SKPs caused C-GAG contraction and increased the mechanical strength of the matrix. Notably, SKPs in C-GAG expressed high levels of genes involved with hair induction. Moreover, ESSs populated with culture-expanded human adult epidermal stem cells (Epi-SCs) and culture-expanded SKPs, but not dermal fibroblasts, regenerated the hair follicle and sebaceous gland after transplantation into wounds in nude mice. Thus, our results indicate that SKPs can serve as hair-inductive cells in ESS in order to enhance its ability in appendage regeneration.

## 2. Experimental Section

### 2.1. Preparation of the C-GAG Matrixes

Acellular C-GAG matrix was prepared by freeze-drying a co-precipitate of type I collagen and chondroitin-6-sulfate, as reported previously [23]. Briefly, collagen (0.5 wt%; Mingrang. Ltd., Sichuan, China) was solubilized in 0.5 M acetic acid and co-precipitated with chondroitin-6-sulfate (0.05 wt%; Sigma, St. Louis, MO, USA). The co-precipitate was mixed (15,000 rpm, 4 °C, 4 h) with a homogenizer (Polytron, Göteborg, Sweden), and was subsequently degassed under centrifugation (3500 rpm, 4 °C, 5 min). The degassed C-GAG suspension was then cast between a custom frame and frozen to −80 °C at a constant rate in an isopropanol (Sigma, St. Louis, MO, USA) bath, and was held at that temperature for 1 h. The frozen casting apparatus was opened, and the C-GAG matrix was freeze-dried (FreeZone^6^Plus, Labconco, Kansas City, MI, USA) for 24 h in order to produce highly porous matrixes. The matrix obtained was cross-linked by dehydrothermal treatment at 140 °C under vacuum for 24 h in a drying oven (Yiheng, Shanghai, China).

### 2.2. Determination of the Pore Structure and Sizes of the C-GAG Matrix

The microstructures of the C-GAG matrix were observed using a LEO 1430 scanning electron microscope (SEM). Cross-sections of the matrix were sputter-coated with gold for 2 min at 15 mA, and were viewed at 20 kV at 250 × and 500 × magnifications. The pore structure of the matrix was examined, and the pore size was determined by NIH Image J. The pore size of at least 50 pores was determined for each matrix.

### 2.3. Mice

C57BL/6 (7 weeks old) and BALB/c nu/nu mice (5 weeks old) were purchased from Guangdong Medical Laboratory Animal Center, Guangzhou, China. C57BL/GFP (green fluorescent protein) mice (6 weeks old) were obtained from Cyagen Biosciences, Guangzhou, China. The animals were maintained in a temperature-controlled environment (20 ± 1 °C) with access to food and water throughout the experiment. All of the animal procedures were performed with the approval of the Ethics Committee of Shenzhen Center for Disease and Prevention. All of the methods were performed in accordance with the relevant guidelines and regulations.

### 2.4. Isolation and Culture of Skin-Derived Precursors

The SKPs were generated from the dorsal back skin of neonatal C57BL/6 or C57BL/GFP mice, and cultured as described [24]. The dorsal back skin was cut into 2 ~ 3 mm^2^ pieces, washed three times in Hanks Balanced Salt Solutions (Gibco, New York, NY, USA), and treated with 0.3% Dispase II (Sigma, St. Louis, MO, USA) for 90 min at 37 °C. The epidermis was manually removed and digested with 0.2% collagenase I (Sigma, St. Louis, MO, USA) for 60 min at 37 °C. The epidermal cells were collected for transplantation. The dermis was digested with 0.3% collagenase I for 30 ~ 40 min at 37 °C. The dissociated cells were cultured in SKP growth medium and Dulbecco’s modified Eagle’s medium/F12, 3:1 (Invitrogen, Waltham, MA, USA) containing B27 (Gibco, New York, NY, USA), 20 ng/mL epidermal growth factor, and 40 ng/mL basal fibroblast growth factor (both from Peprotech, Rocky Hill, NJ, USA).

Human foreskins were used for the establishment of the Epi-SCs, as previously described [25]. Briefly, foreskins from healthy donors were harvested with written informed consent; the procedure was approved by the Ethics Committee of Peking University Shenzhen Hospital, and the method was performed in accordance with the relevant guidelines and regulations. Then, Epi-SCs were isolated on the basis of their high adhesive properties, and were cultured in CnT-07 progenitor cell-targeted epidermal keratinocyte medium (CellnTec, Bern, Switzerland).

### 2.5. Cell Viability and Proliferation in the C-GAG Matrix

The C-GAG matrix was punched into round sheet samples (10 mm diameter). The samples were placed into wells of non-treated 24-well plates and sterilized for 30 min with UV radiation. Passage 2 SKPs were harvested and used for the in vitro experiments. SKPs were seeded into each matrix at a density of 1.0 × 10^5^ cells/cm^2^. After incubation for 2 h, 500 μL SKP culture medium was added for each matrix. The medium was changed every two days. The matrixes with the cells were cultured (at 37 °C, 5% CO_2_) for up to 10 days, and subjected to analyses as follows.

The cell viability was determined using the LIVE/DEAD Viability/Cytotoxicity kit for mammalian cells (Invitrogen, Waltham, MA, USA). C-GAG matrixes loaded with cells were washed three times with phosphate-buffered saline (PBS), and were subsequently covered with a solution of ethidium homodimer-1 and calcein acetoxymethyl ester, both at an optimized concentration of 2 mM. After incubation at 37 °C for 30 min, the samples were rinsed with PBS and visualized under a confocal laser scanning microscope (FV1000, Olympus, Tokyo, Japan).

The number of cells was examined by the fluorometric quantification of the amount of cellular DNA using a PicoGreen dsDNA Quantitation kit (Invitrogen, Waltham, MA, USA). Briefly, the cultured samples were treated in papain digestion (Sigma, St. Louis, MO, USA) buffer containing 0.1 mg/mL papain, 5 mM sodium citrate, 5 mM cysteine hydrochloride, and 5 mM ethylene diamine tetraacetic acid (EDTA) pH 7.5 at 60 °C for 2 h. In total, 10 μL cell lysate was mixed with 200 μL DNA binding fluorescent dye solution (1 μL PicoGreen reagent in 200 mL 1 × TE buffer). The fluorescent intensity of the mixed solution was measured on a microplate reader (Tecan, Zürich, Switzerland).

The cell proliferation and morphology studies in the C-GAG matrixes were conducted using the confocal microscopic technique. SKPs from C57BL/GFP mice were used. Briefly, matrixes with cells were harvested and washed three times with PBS (pH 7.4). The cells were fixed for 10 min by incubating in 4% paraformaldehyde (PFA, Sigma, St. Louis, MO, USA). For the cell proliferation, the fluorescence images were obtained using a confocal laser scanning microscope (FV1000, Olympus, Japan).

The cell morphology was observed using SEM. Briefly, matrixes with cells were fixed in 2.5% (*w/v*) glutaraldehyde at 4 °C for 30 min. After fixation, the samples were washed gently in double-deionized water. Then, the matrixes were quickly frozen in liquid nitrogen and transferred to a freeze dryer. The samples were sputter-coated with gold and platinum alloy, and were examined at 5 kV.

### 2.6. Analysis of the C-GAG Matrix Contraction Caused by SKPs

In order to determine the extent of the SKP-mediated matrix contraction, C-GAG matrixes seeded with SKPs were photographed on days 0, 2, 4, 7 and 14, and the images were analyzed using NIH image J. The percentages of contraction of the matrixes were calculated by determining the change in the area of the matrixes at each time point with respect to the original area; the original area of the matrix before seeding with the SKPs was considered to be 100%. C-GAG matrixes without cells were used as controls.

### 2.7. Biomechanical Testing of the C-GAG Matrixes with SKPs

The mechanical properties of the acellular and cellular C-GAG scaffolds were assessed via a tensile tester [26]. Briefly, acellular scaffolds were re-hydrated, placed on Conformant 2 (Smith&Nephew, Andover, MA, USA) sheet for ease of handling, and were cut into specimens with a length of 40 mm and width of 7 mm. Then, the samples were mounted into the clamp of a Labsans tensile tester model and tested to break at a strain rate of 50 mm/min. The samples were strained to failure, and the data from the samples which did not break within the gauge length were discarded. The cellular scaffolds were cultured for 10 days, and were evaluated following the same protocol as the acellular scaffolds. The percent elongation, ultimate tensile strength (UTS), and stiffness values were measured. The average values were calculated from six samples (*n* = 6).

### 2.8. Real-Time PCR Analysis

The total RNA of the samples was extracted by TRIzol (Invitrogen, Waltham, MA, USA), and the RNA yield was quantified using a Nanodrop (ThermoFisher Scientific, Waltham, MA, USA). The first-strand cDNA was synthesized by reverse transcription with Superscript II reverse transcriptase (Invitrogen, Waltham, MA, USA). The cDNA was stored at −80 °C. The real-time PCR was performed using a SYBR Green Real-Time PCR Master Mix (Toyobo, Osaka, Japan) on an ABI 7300 QPCR System. Glyceraldehyde-3-phosphate dehydrogenase (GAPDH) was used as an endogenous housekeeping gene. The relative expression of each target gene was examined using the 2^−ΔΔCt^ method. The primer sets are shown in Supplementary Material Table S1.

### 2.9. Alkaline Phosphatase Activity

The alkaline phosphatase (AP) activity was examined as previously described [27]. The AP staining was performed using an AP Staining Kit (Beyotime Biotechnology, Jiangsu, China) according to the manufacturer’s protocol. Briefly, the SKPs in a C-GAG matrixes were rinsed with PBS, and fixed in 4% PFA for 30 min at room temperature. After washing, the staining buffer was added to the samples and incubated at 37 °C for 2 h. After washing, the samples were observed under a light microscope. The AP activity of SKPs in a C-GAG matrixes was measured using an AP Assay Kit (Beyotime Biotechnology, Jiangsu, China). The matrixes with SKPs were rinsed twice with cold PBS and lysed in a lysis buffer containing 0.1% Triton X-100 (Sigma, St. Louis, MO, USA). After centrifugation at 4 °C for 10 min, the upper aqueous phase was used to measure the AP activity. The absorbance was read at 405 nm using a microplate reader (BioTek, Shoreline, WA, USA) to determine the level of AP. The values were normalized to the amount of total intracellular protein determined using a Protein Assay Kit (Thermo, Waltham, MA, USA).

### 2.10. Hair Follicle Regeneration Assay

BALB/c nu/nu mice (5–7 weeks old) were anesthetized by an intraperitoneal injection of sodium pentobarbital (50 mg/kg). A 10 mm diameter, full thickness skin wound was created on the back of each mouse with a skin biopsy punch, as previously described [28]. The ESS was prepared by seeding a mixture of 5 × 10^6^ SKPs with 3 × 10^6^ neonatal mouse Epi-SCs or culture-expanded human Epi-SCs in a C-GAG matrix. The ESS was incubated for 2 h in a 37 °C, 5% CO_2_ tissue culture incubator, and was implanted into the excisional wound. The wound was then covered with Tegaderm (3M, St.Paul, MN, USA) transparent dressing, which was further covered with a self-adhering elastic bandage. After 3 weeks, the mice were sacrificed, the numbers of hairs were counted under a dissecting microscope, and wound tissue samples were harvested for histological analysis. The hair shafts were analyzed under light microscopy and SEM. For the SEM observations, the hair shafts were dehydrated in 100% ethanol. After coating with platinum, the samples were examined using an LEO 1430 SEM.

### 2.11. BrdU Labeling and Detection

Human Epi-SCs were incubated with 10 μM 5-Bromo-2’-Deoxyuridine (BrdU) (Sigma, St. Louis, MO, USA) for 24 h at 37 °C and 5% CO_2_, and then transplanted into wounds in mice. Their skin tissues were harvested, and 10 μm thick cryostat section samples were obtained. The samples were treated with 2 N HCl for 30 min, and washed with 0.1M borate buffer (pH 8.5) for 15 min and then with PBS for 3 times. After blocking, the sections were incubated with an anti-BrdU antibody (Biolegend, San Diego, CA, USA, 1:100) at 4 °C overnight, and were detected with a fluorescence-conjugated secondary antibody. The samples were examined under a confocal laser scanning microscope (FV1000, Olympus, Japan).

### 2.12. Histological Analysis

The tissues were fixed with 4% PFA for 12 h, dehydrated in ethanol solutions, embedded in paraffin, sectioned in 5 µm thickness, and mounted on microscope slides. The slides were then stained with hematoxylin and eosin, and were viewed under light microscopy.

### 2.13. Immunofluorescence Analysis

The skin tissues were fixed with 4% PFA for 12 h, dehydrated in sucrose solutions, embedded in optimal cutting temperature (OCT) compound and sectioned (10 µm thick). The samples were washed with PBS and blocked with 5% bovine serum albumin (BSA)/PBS containing 0.2% Triton-X 100 (Sigma, St. Louis, MO, USA) at 37 °C for 1 h. The samples were incubated with primary antibodies in 1% BSA/PBS at appropriate concentrations at 4 °C overnight: keratin (K)14(Biolegend, San Diego, CA, USA, 1:100), K1(Biolegend, San Diego, CA, USA, 1:100), Ki67 (ThermoFisher Scientific, Waltham, MA, USA, 1:200), and CD49f-biotin (Biolegend, San Diego, CA, USA, 1:150). After washing, the samples were detected with fluorescence-conjugated secondary antibodies. The nuclei were stained with 4′,6-diamidine-2′-phenylindole dihydrochloride (DAPI). The samples were examined under a confocal laser scanning microscope (FV1000, Olympus, Japan).

### 2.14. Statistical Analysis

All of the values are expressed as mean ± SD. One-way ANOVA was used for multiple group comparisons. A probability (*p*) value < 0.05 was considered significant.

## 3. Results

### 3.1. Characterization of the C-GAG Matrix

**Figure 1:**
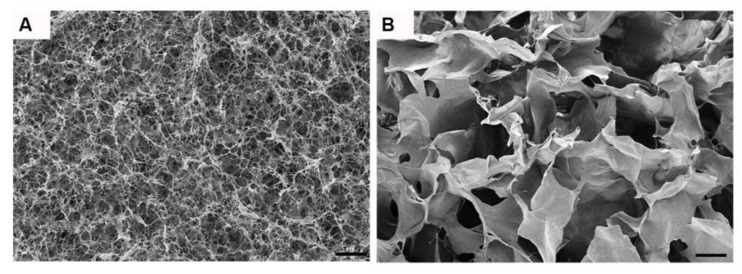
Pore size of the C-GAG matrix. The pore size of the dehydrothermally cross-linked C-GAG matrix was determined by SEM. (**A**) Superficial structure. (**B**) Internal structure. 200 ×. Scale bars: 50 μm.

Acellular C-GAG matrixes were prepared by freeze-drying a coprecipitate of type I collagen and chondroitin-6-sulfate, and were subsequently cross-linked by dehydrothermal treatment. The resulting C-GAG matrixes were examined by scanning electron microscope (SEM), and showed heterogeneous pore structures and sizes. Superficial reticulated fibers were observed (Figure 1A). Transverse sections of the C-GAG showed an internal structure of randomly-oriented pores separated by thin collagen struts (Figure 1B). The average pore size was 52 ± 17 μm.

### 3.2. SKPs Are Viable in the C-GAG Matrix

**Figure 2:**
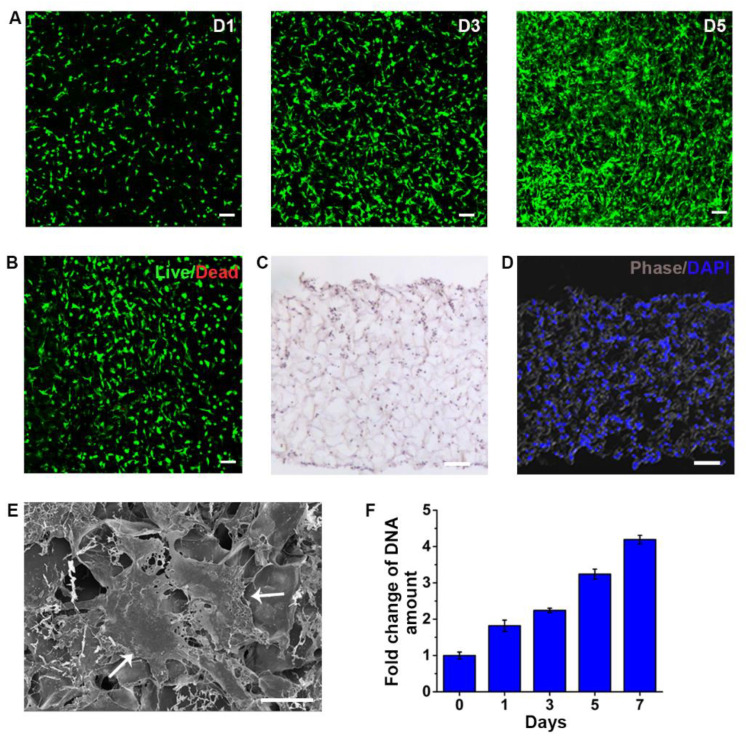
Proliferation of SKPs in the C-GAG matrix. (**A**) SKPs expressing GFP in a C-GAG matrixes after culture for 1 (left), 3 (middle), and 5 (right) days, respectively. (**B**) Cell viability. Live/dead staining of the SKPs after incubation for 3 days. (**C**,**D**) SKPs distribution in C-GAG. After incubation for 10 days, the C-GAG matrixes with SKPs were sectioned and analyzed by HE and DAPI staining. (**E**) The SKPs’ morphology and their interactions with the matrix was assessed by SEM analysis after culturing for 10 days. Arrows: SKPs. (**F**) SKPs’ growth. The numbers of cells in the C-GAG matrixes were quantified by measuring the amounts of DNA. Scale bars: 50 μm.

SKPs expressing GFP in passage 2 were seeded on a C-GAG matrix and cultured. The cells were assayed at different time points to determine their survival and proliferation. Fluorescence microscopy showed that the cells spread with processes extending into the matrix (Figure 2A). In accordance with this, a cell proliferation analysis by measuring the quantity of DNA extracted from the matrix at 1, 3, 5 and 7 days indicated that the number of SKPs increased progressively (Figure 2F). The cell viability analysis at 1, 3, 5, 7 and 10 days using a live/dead kit indicated that—at all time points over 90%—the cells were viable (Figure 2B and Supplementary Figure S1). After 10 days, the histological analyses of the matrix indicated that SKPs had migrated into the matrix and proliferated, resulting in an even distribution of the cells in the matrix (Figure 2C,D). The SEM analysis showed the delicate attachment of the cells to the matrix via their processes (Figure 2E).

### 3.3. SKPs Mediate C-GAG Matrix Contraction

**Figure 3:**
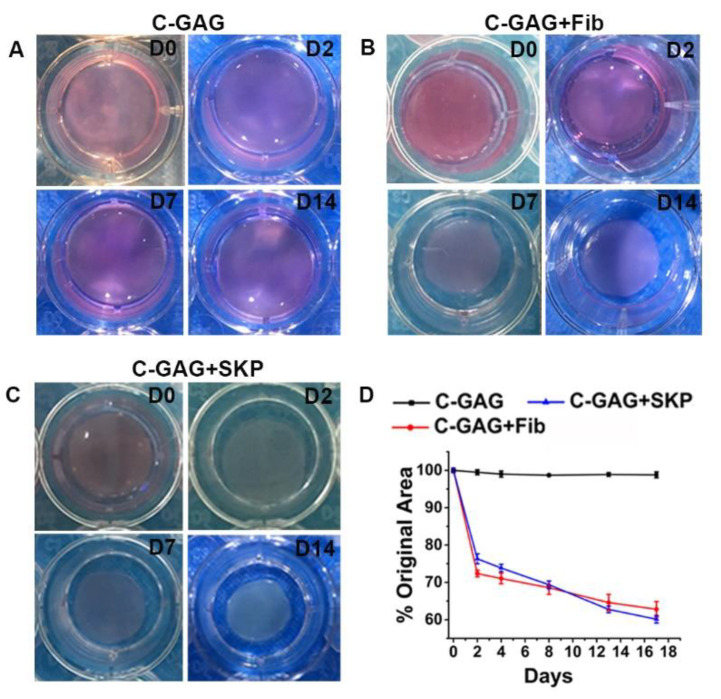
SKP-mediated C-GAG matrix contraction. (**A**–**C**) Digital photographs of SKPs-populated C-GAG matrixes were taken at 0, 2, 7 and 14 days of incubation. (**A**) Matrix without cells (control). (**B**) Matrix with fibroblasts. (**C**) Matrix with SKPs. (**D**) Quantification of the matrix contractions using NIH Image J. The values represent the mean ± SD (*n* = 3).

An important part of wound healing is wound contraction, in which the motile activity of fibroblasts plays a critical role. In order to determine whether the SKPs were functional and could contribute to wound contraction, 2 × 10^5^ SKPs were seeded in a C-GAG matrixes (round sheet, 16 mm in diameter), and the contraction was measured at different time points. The size of the matrixes seeded with SKPs declined progressively in 2 weeks, with a dramatic reduction by 24% ± 3% at 48 h, similar to the matrixes seeded with dermal fibroblasts, while the size of the matrixes without cells remained unchanged (Figure 3).

### 3.4. SKPs Increase the Strength of C-GAG Matrix

**Figure 4:**
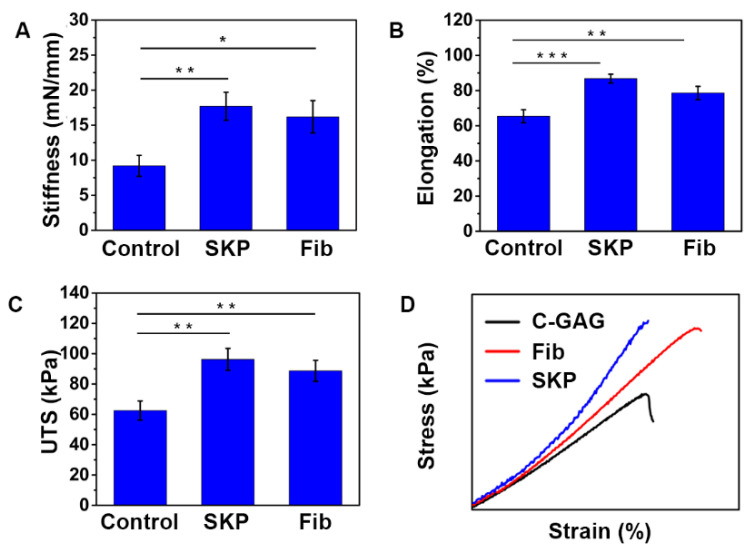
Biomechanical properties of SKP-populated C-GAG matrixes. The biomechanical properties of acellular and SKPs or dermal fibroblast-populated C-GAG matrixes were assessed after 10 days of culture using a tensile tester. (**A**) Stiffness. (**B**) Elongation. (**C**) UTS. (**D**) Representative stress–strain curve of C-GAG matrixes. The values represent the Mean ± SD (*n* = 6). * *p* < 0.05; ** *p* < 0.01; *** *p* < 0.001.

The mechanical properties of the C-GAG matrix are critical for preserving the structural integrity and functionality during both in vitro long-term culture and in vivo implantation. As shown above, the SKPs contracted and remodeled the C-GAG matrix (Figure 3); it is necessary to investigate the mechanical performance of the SKPs-seeded C-GAG matrix. C-GAG matrixes seeded with SKPs (2.0 × 10^5^ cells/cm^2^) were cultured for 10 days and subjected to biomechanical analyses. Before the test, the C-GAG matrix was cut into rectangle-shaped pieces (gauge length of 40 mm and width of 7 mm). The stiffness, UTS, and elongation of a hydrated acellular C-GAG matrix was 9.2 ± 2.1 mN/mm, 62.5 ± 9.3 kPa and 65.4 ± 5.7%, respectively (Figure 4A–C). SKP-populated matrixes showed greater stiffness, UTS, and elongation to 17.7 ± 2.9 mN/mm, 90.3 ± 10.2 kPa and 86.8 ± 6.5%, respectively (*p* < 0.01). However, the average stiffness of the SKP-populated C-GAG matrixes was similar to dermal fibroblast-populated C-GAG matrixes (16.2 ± 3.3 mN/mm, *p* > 0.05). In addition, the stress–strain curves indicated a similar mode of deformation between SKP-populated C-GAG matrix and fibroblast-populated C-GAG matrix (Figure 4D).

### 3.5. SKPs Express Hair Follicle-Inductive Genes in the C-GAG Matrix

**Figure 5:**
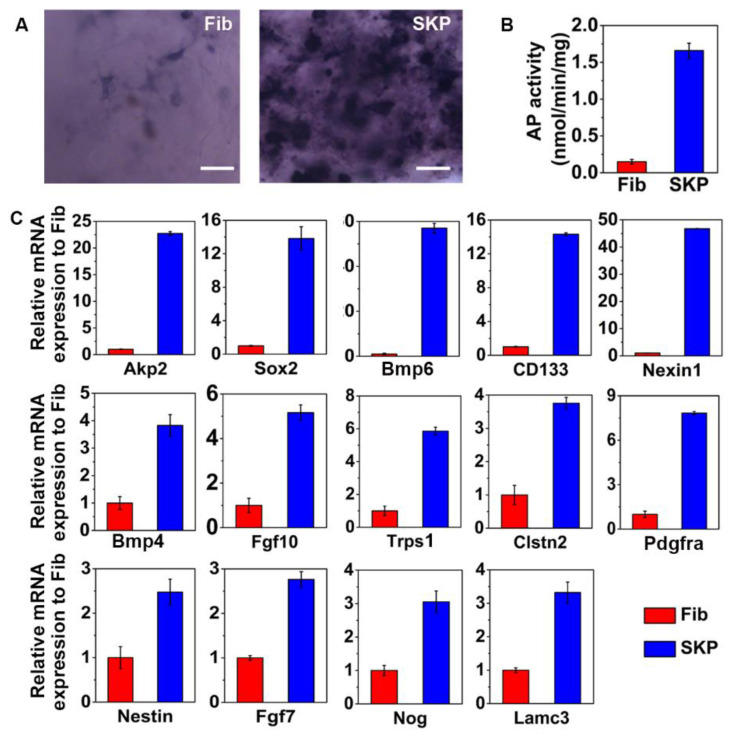
Gene expressional analysis of SKPs in a C-GAG matrixes. (**A**,**B**) The AP activity of SKPs and fibroblasts (Fib) in a C-GAG matrix after 3 days of culture was determined using an AP kit. (**A**) The representative images are shown. (**B**) The AP activity was quantified. Scale bars: 100 μm. (**C**) Real-Time PCR analysis of SKPs and fibroblasts in a C-GAG matrix for 3 days for their expression of genes involved with hair genesis. This is a representative result of three independent experiments.

As the expression level of AP is largely related to the hair-inductive ability of DP cells [29], and SKPs have a similar property to DP cells in inducing hair genesis [14], we examined the AP expression of SKPs in the C-GAG matrix. In total, 2.0 × 10^5^ SKPs or dermal fibroblasts (control) were seeded on C-GAG matrixes (10 mm in diameter) and incubated for 3 days. An AP stain showed that the SKP-populated matrix exhibited more intensive reactions than the fibroblast-populated matrix (Figure 5A). Quantitative analysis indicated a much higher level of AP activity in SKPs (Figure 5B). Genes known to be involved in the hair follicle-inductive property of DP cells—namely DP signature genes—were examined in SKP- or fibroblast-populated matrixes by Real-Time PCR analysis. In consistence with the AP stain results, the expression of *Akp2* in SKPs was 23-fold higher than that in fibroblasts. In addition, the expression levels of *Sox2, Bmp6, CD133* and *Nexin1* in the SKPs were more than 10-fold higher than those in fibroblasts. Moreover, SKPs also showed higher expression levels of *Bmp4*, *Fgf10, Trps1, Clstn2, Pdgfra, Nestin, Fgf7, Nog* and *Lamc3* compared to fibroblasts (Figure 5C). These results are highly suggestive of the hair-inductive potential of SKPs in a C-GAG matrix.

### 3.6. SKPs in a C-GAG Matrix Induce de novo Hair Genesis

**Figure 6:**
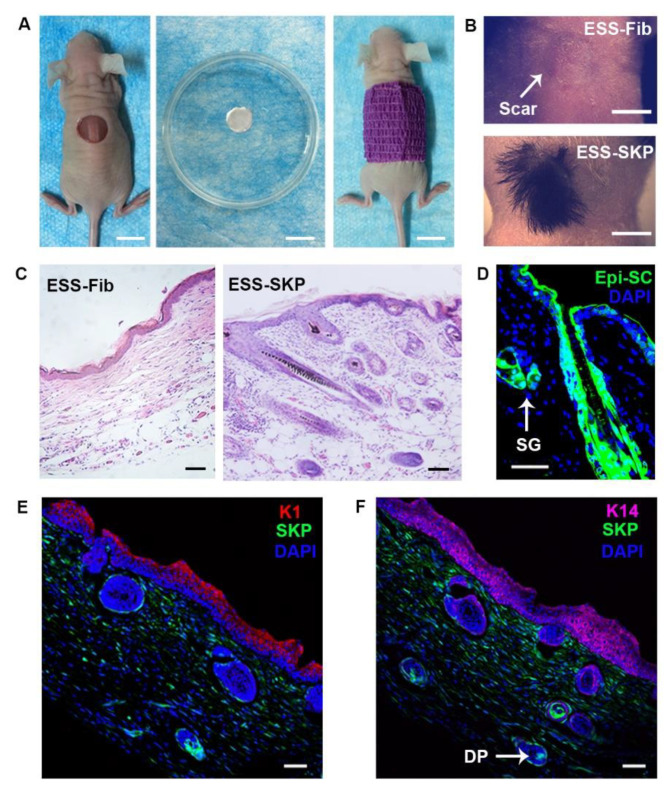
SKP-induced hair genesis in a C-GAG matrix. (**A**) The ESS was prepared by seeding a mixture of culture-expanded murine SKPs (or dermal fibroblasts as a control) and neonatal Epi-SCs into a C-GAG matrix (named as ESS-SKP or ESS-Fib), and was incubated for 2 h (middle). The ESSs were then transplanted into full-thickness wounds in nude mice (right). The wounds were finally dressed with Tegaderm and bandage (left). (**B**) Three weeks later, the wounds which received ESS-SKP (lower) healed with black hairs grown, while the wounds which received ESS-Fib (upper) healed without hair grown. Scale bars: 10 mm. (**C**) Histological analysis showed that the wounds which received ESS-SKP had densely-populated hair follicles and sebaceous glands in the dermis, while the wounds which received ESS-Fib showed fibrotic structure in the dermis (H&E stain). Scale bars: 50 μm. (**D**–**F**) Fluorescence analysis of the ESS-SKP treated wound. (**D**) The newly-formed hair follicles and sebaceous glands were derived from the donor Epi-SCs expressing GFP. SG: sebaceous glands. Scale bar: 50 μm. (**E**,**F**) The donor SKPs contributed to spindle-shaped cells in the dermis and the DP in the hair follicle. The neoepidermis showed a ‘stratified’ epidermis, revealing a normal pattern of differentiation. Scale bars: 50 μm.

Our previous study indicated that Epi-SCs derived from the basal layer epidermis of mice and humans were capable of forming de novo hair follicles, even after culture expansion [30]. In this study, we examined the hair-inductive property of SKPs for Epi-SCs in a C-GAG matrix. The ESS was prepared by seeding 5 × 10^6^ culture-expanded murine SKPs (or dermal fibroblasts as a control) in combination with 3 × 10^6^ neonatal mouse Epi-SCs into a round C-GAG matrix (10 mm in diameter), and was named as ESS-SKP (or ESS-Fib). After culturing for 2 h, the ESS was implanted into full-thickness wounds in nude mice (Figure 6A). Three weeks later, black hairs were generated from the wounds which received ESS-SKP, but not ESS-Fib (Figure 6B). The regenerated hairs lasted as the recipient mice lived and underwent cyclic changes.

The histological analysis revealed densely-populated hair follicles and sebaceous glands in the dermis of the newly-formed skin treated with ESS-SKP (Figure 6C). In order to track the contribution of transplanted cells in skin regeneration, SKPs or Epi-SCs from C57BL/GFP mice were used. The immunofluorescence analysis of the wound tissue indicated that the regenerated epidermis, hair follicles and sebaceous glands were largely derived from the implanted Epi-SCs (Figure 6D), while the donor-derived SKPs contributed to the DP in the neogenic hair follicles and numerous cells in the newly formed dermis. The immunofluorescence analysis of the expression of K1 and K14 in the regenerated epidermis showed a ‘stratified’ structure, similar to that in the normal epidermis (Figure 6E,F).

**Figure 7:**
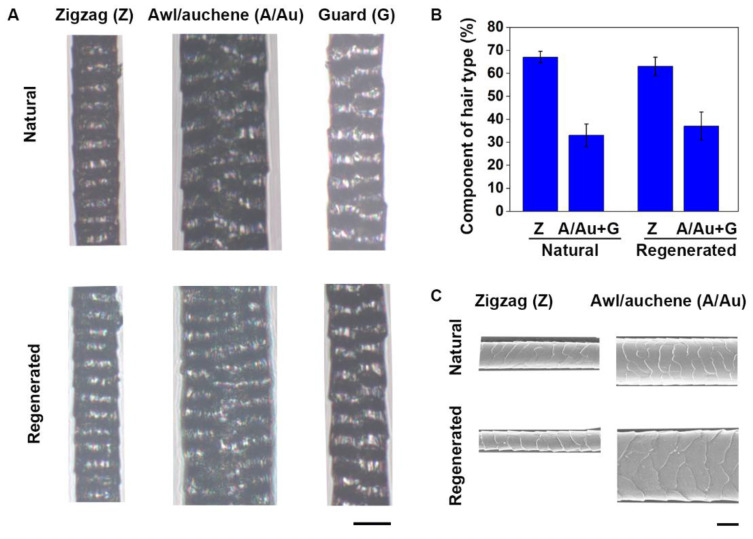
Analysis of the hairs regenerated by ESS. ESS consisting of cultured mouse SKPs and neonatal mouse epidermal cells in C-GAG were transplanted into excisional wounds in nude mice. The hairs regenerated from the grafts were analyzed 21 days after transplantation, and were compared with natural hairs collected from the back of wild type mice. (**A**) Microscopic observation showed that the regenerated hairs included zigzag (Z), awl/auchene (A/Au), and guard (G) hairs, similar to natural mouse hairs. Scale bar, 25 μm. (**B**) The amount of different types of regenerated hairs were counted and compared to that of natural mouse hairs. Hairs from six grafts were analyzed, and 100 hairs per graft were examined. (**C**) The hair shafts were analyzed under SEM. Scale bar, 25 μm.

In mouse skin, there are several distinct hair follicle types that differ in length, thickness, and in the presence or absence of kinks in the hair shaft. For hair regenerative therapy, it is critical to consider whether neogenic hair follicles can regenerate normal inherent traits and physiological functions, such as hair shaft types and qualities. We further examined the structure of the hair shafts of the follicles regenerated from grafts of ESS consisting of SKPs and neonatal mouse epidermal cells in C-GAG under a microscope, and compared them with hairs collected from the back of adult wild-type C57 mice. We found that, similar to the natural hairs of C57 mice, the regenerated hairs consisted of all types of pelage hairs—e.g., zigzag, awl/auchene and guard—and had a central cavity, corresponding to a ladder-like structure observed under light microscope (Figure 7A,B). Moreover, under SEM, the regenerated hairs exhibited the typical structure of natural hairs, including the medulla and cortex (Figure 7C). These results indicate that our ESS regenerate normal hairs, in both hair type and structure.

**Figure 8:**
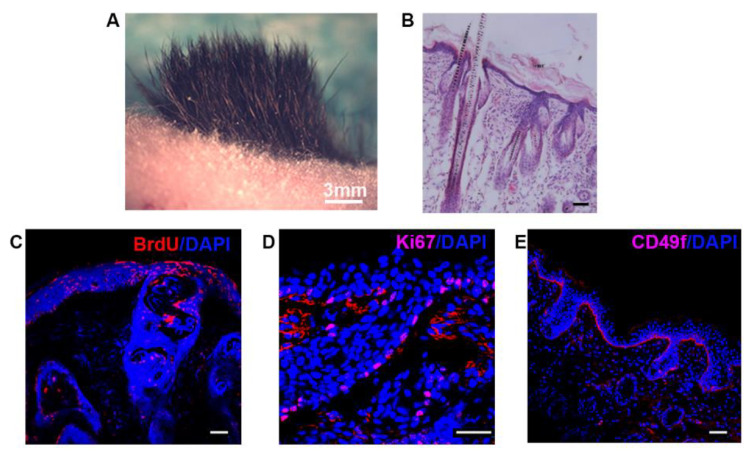
SKPs induced human Epi-SCs to form hair follicles in a C-GAG matrix. The C-GAG matrix was seeded with a mixture of culture-expanded SKPs and adult human Epi-SCs (derived from foreskin), and was implanted into excisional wounds in nude mice. (**A**) Three weeks later, hairs generated at the wound site. Scale bar: 3 mm. (**B**) Histological analysis of the wound site showed hair follicles and sebaceous glands in the dermis. (**C**) When the human Epi-SCs were pre-labeled with BrdU before transplantation, fluorescence analysis using an antibody against BrdU revealed the presence of human cells in the newly-formed epidermis and hair follicles. (**D**) The Ki67^+^ stain indicated that many cells in the neogenic hair follicles and epidermis were dividing. (**E**) Staining with an antibody against CD49f detected positive cells in the basal layer epidermis, suggesting the formation of a long-term renewable structure by human Epi-SCs. Scale bars: 50 μm.

Next, we determined whether culture-expanded SKPs in a C-GAG matrix were able to induce culture-expanded human Epi-SCs to generate hairs. Similarly, the ESS was obtained by seeding a mixture of five million murine SKPs (in passage 2) and three million human Epi-SCs derived from the epidermis of adult human foreskin in passage 2 into a round C-GAG matrix (10 mm in diameter). For the in vivo tracking, the Epi-SCs were labeled with BrdU; the Epi-SCs of three individuals (age 15–25 years) were used to prepare the ESSs. The ESSs were implanted into excisional wounds in nude mice. Three weeks after transplantation, densely-populated black hairs were found at the transplantation sites of all three ESSs seeded with the human Epi-SCs of three donors, respectively (Figure 8A). Histological analysis revealed densely-populated hair follicles and sebaceous glands in the neogenic skin (Figure 8B). The cells in the neogenic epidermis and hair follicles were largely positive for BrdU (Figure 8C), suggestive of human origin. In addition, many cells in the newly-generated structures—particularly the basal layer of the epidermis and the outer layer of the hair follicle—expressed Ki67 (Figure 8D), indicating the active proliferation of the epithelial cells. Moreover, antibody-targeting CD49f—a marker for Epi-SCs—detected positive cells in the basal layer epidermis (Figure 8E), implying the formation of a long-term renewable stem cell pool by human Epi-SCs.

## 4. Discussion

To develop an ESS capable of fully restoring the structure and function of the injured skin has been the objective in tissue engineering. Current cellularized ESS consisting of culture-expanded autologous epidermal cells and dermal fibroblasts is able to regenerate the epidermis, but not its appendages [31,32,33]. Several reasons may contribute to the failure of appendage regeneration, such as the lack or insufficiency of stem cells, appendage-inductive cells, and/or appropriate scaffolds. Our previous study and others suggest that the lack of hair follicle-inductive cells is likely the major cause for the deficient regeneration of the hair follicle by ESS [27,30]. Firstly, in a hair follicle recombination assay, the transplantation of hair follicle stem cells plus DP cells, but not dermal fibroblasts, formed de novo hair follicles. Secondly, the transplantation of Epi-SCs derived from the epidermis of mice or humans plus SKPs also formed de novo hair follicles. In this study, we show that a combination of culture-expanded Epi-SCs and SKPs in C-GAG successfully regenerated the hair follicles and sebaceous glands in mice. Our results indicate that the C-GAG matrix provides an appropriate environment for SKPs to proliferate and excise their hair induction function. SKPs form intensive interactions with the scaffold and continuously proliferate. Importantly, SKPs vigorously express the genes involved with hair induction, such as *Akp2, Bmp6, Sox2* and *CD133*.

The pore structure is an essential consideration in the development of scaffolds for tissue engineering [34,35,36,37]. In this study, the C-GAG matrix was fabricated using a freeze-drying (lyophilisation) process whereby a constant cooling rate (1 °C/min) technique was used to produce scaffolds with a homogenous pore structure. SEM analysis revealed that the cross-linked C-GAG matrix had a mean pore size of 52 ± 17 μm (Figure 1), an optimal size known to support the survival, growth, and many other activities of cells being seeded inside [38,39,40]. Previous works indicated that smaller pores restrict the migration of cells into the scaffolds [41,42]. In this study, the SKPs infiltrated into the C-GAG matrix after topical seeding, and were subsequently distributed evenly throughout the scaffold. These results indicate that the C-GAG matrix is potent in supporting SKPs to induce hair follicle regeneration.

SKPs cause remodeling to the C-GAG matrix. We found that SKP-populated C-GAG matrixes contracted progressively upon culturing, similar to DP cells [43]. This is likely caused by the active migration of SKPs through the collagen fibrils in the matrix. In addition, SKP-populated C-GAG matrixes showed increased stiffness and UTS, which are appropriate properties for the easy handling of the ESS in future clinical uses.

The development of ESS capable of restoring the structure and function of the skin requires competent cells and appropriate scaffolds. In this study, we showed that an ESS formed by seeding a mixture of culture-expanded SKPs and Epi-SCs into a C-GAS matrix could regenerate a complete skin structure with the neogenesis of the hair follicles and sebaceous glands after transplantation into wounds in mice. This novel ESS promises an improved therapeutic potential for burn injuries.

## Data Availability

All data presented in this study are available upon reasonable request from the corresponding author.

## Supplementary Materials

Supplementary materials can be found at https://www.mdpi.com/article/10.3390/biomedicines9040400/s1. Table S1: Primers used for the Real-time PCR. Figure S1: SKP survival. Cell viability analysis at 1, 3, 5, 7 and 10 days using a live/dead kit indicated that, at all time points, over 90% of the cells were viable.
