# Supplementary material for: Engineered Skin Substitute Regenerates the Skin with Hair Follicle Formation
Source: Biomedicines. 2021 Apr 8;9(4):400. doi: 10.3390/biomedicines9040400 (PMC8068101; doi:10.3390/biomedicines9040400)
Supplement: Supplementary file 1 [file biomedicines-09-00400-s001.pdf]

Engineered skin substitute regenerates the skin with hair follicle formation

Table S1. Primers used for Real-time PCR.

Gene	Sense	Antisense
Akp2	TCGGAACAACCTGACTGACCC	CTGCTTGGCCTTACCCTCATG
CD133	CATTTGCCTCTACCCTGGAAGC	ATGCTGGTGGATGGCTCTTATATTC
Bmp4	CAGGGAACCGGGCTTGAG	CTGGGATGCTGCTGAGGTTG
Bmp6	TGTGGTGACTCGGGATGGAC	ATGAAGGGCTGCTTGTCGTAAG
Nexin1	ATTGCAAGGTCATCACCTCCC	AGTCGTCTTGGCTTTGCGTG
Nestin	GGTTCCCAAAGAGGTGTCCG	CAGCAAACCCATCAGACTCCC
Sox2	TCCATGGGCTCTGTGGTCAAG	TGATCATGTCCCGGAGGTCC
Pdgfra	ACGCATGCGGGTGGACTC	GATACCCGGAGCGTGTCAGTTAC
Nog	AAACAGCGCCTGAGCAAGAAG	GAACACTTACACTCGGAAATGATGG
Fgf7	AGCGGAGGGGAAATGTTCG	TCCAGCCTTTCTTGGTTACTGAGA
Fgf10	ATTTCCCCCTGTATGCATCCTAAC	TTCCCACGGAGGCAGAACTC
Clstn2	GAGGCTGCCCAACCTACCATT	CTGGGAACAAACAGGCTTCTCAC
Trps1	AGCCCAGGGTTCATTGACTAAAAG	AAGCCAGGCACATGACTCAAGTAG
Lamc3	GGCCCTGGACCCACTATGC	GTGGGGCCACAAGACCAGTC

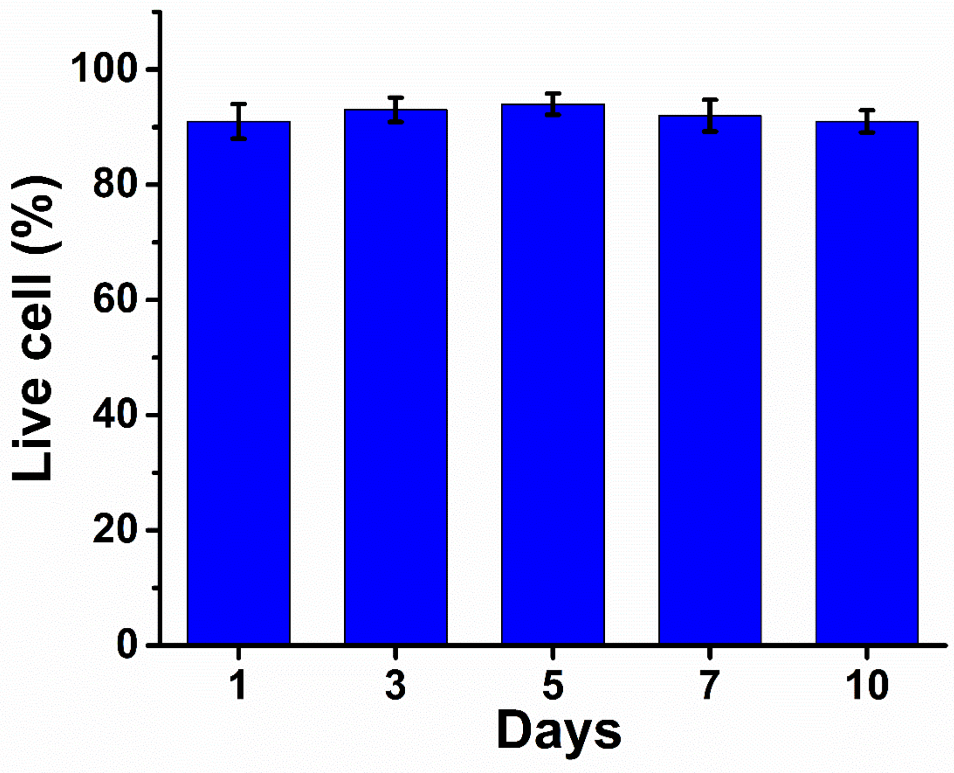

Figure S1. SKP survival. Cell viability analysis at 1, 3, 5, 7 and 10 days using a live/dead kit indicated that at all time points over 90% cells were viable.
